# Does vaginal bacterial colonization contribute to preterm birth in women with asymptomatic shortened cervix?

**DOI:** 10.1007/s00404-024-07397-2

**Published:** 2024-04-05

**Authors:** J. Steetskamp, M. Zander, V. Laufs, T. Elger, A. Hasenburg, C. Skala

**Affiliations:** 1 Department of Obstetrics and Gynecology, Mainz University Medical Center, Langenbeckstr. 1, 55131 Mainz, Germany; 2. Josefs-Hospital Wiesbaden, Beethovenstraße 20, 65189 Wiesbaden, Germany

**Keywords:** Vaginal bacterial colonization, Pregnancy, Preterm delivery, Asymptomatic shortened cervix, *Ureaplasma*

## Abstract

**Purpose:**

The aim of this study is to describe the typical microbial spectrum and the influence of distinct vaginal infections on preterm birth in pregnancies affected by cervical incompetence.

**Methods:**

327 patients were admitted because of asymptomatic shortening of the cervix in the second and third trimester of pregnancy. Clinical data such as age, cervical length, gestational age at admission and at delivery and vaginal microbiologic findings were collected and analyzed.

**Results:**

The spectrum of germs in the vagina revealed seven different distinct species; the most common bacteria were *Ureaplasma* spp. and *E. coli*. In 327 included patients, 217 revealed a bacterial colonization, 110 did not. Most common bacteria in women with preterm birth before 34 weeks were *Ureaplasma* spp., while *E. coli* was most common in women undergoing preterm birth after 34 weeks. Nevertheless, the rates of occurrence of these bacterial taxa were not significantly different between who underwent preterm birth to those who did not.

**Conclusions:**

This study gives an overview over the vaginal bacterial colonization in pregnant women with cervical incompetence. The clinical relevance of vaginal bacterial colonization remains unclear.

## What does this study add to the clinical work

**Table:** 

This paper gives an overview over the bacterial colonization in pregnancy with a shortening of the cervix and the kinds of germs which are involved. Bacterial colonization with bacterial taxa known to be potentially contributing to preterm birth is common, but the pathogenetic relevance in cervical incompetence remains unclear.	

## Introduction

Preterm delivery has a great impact on perinatal mortality and morbidity. During the last years, the incidence of preterm delivery in Germany remains stable at around 8%. The incidence of a delivery before 32 weeks of pregnancy is 1.42% [1]. The etiology of preterm delivery is multifactorial. One reason for preterm delivery is a preterm maturation of the cervix uteri, which leads to a cervical incompetence. The maturation of the cervix is a coordinated biochemical process, which is influenced by prostaglandins, estrogen and proteases. Inflammations can accelerate this process [2]. Although many kinds of vaginal bacterial colonization do not cause any symptoms, it must be considered that a vaginal infection can cause inflammatory processes. In one-third of preterm deliveries, intraamnial infections are recorded [3]. Therefore, vaginal smear tests in patients at risk of preterm birth are established obstetrical practice. As vaginal colonization is common, it cannot be seen as a pathologic finding per se. There remains the question, which forms of bacterial colonization may be causal for cervical shortening and, subsequently, have to be treated by antibiotics. Antibiotic treatment may have great impact on the development of resistant bacterial strains and disturbances of the physiologic vaginal flora. In this study, we evaluated the occurrence of potential pathogenic vaginal germs in women with a shortening of the cervix and compared the frequency of vaginal colonization in women undergoing preterm birth to those who did not.

## Methods

### Clinical data

Data were acquired by retrospective analysis of all singleton cases with asymptomatic short cervix of less than 25 mm in the second trimester until 33 weeks of gestation admitted to the obstetric department of Mainz university medical center during a 6-year-period (2011–2016). All patients were followed up until delivery. Women admitted with preterm labor or PPROM were excluded. The following data were collected: age, parity, cervical length, gestational age at admission, and gestational age at delivery.

### Microbiologic analysis

At admission, vaginal microbial smears were taken using a sterile swab (eSwab, Copan, Bresica, Italy). Vaginal bacterial colonization was identified by means of bacterial culture, except for *Ureaplasma* spp. and *Mycoplasma* spp., which were identified by PCR testing. In cases of bacterial colonization, antibiotic treatment took place based on the antibiogram. As *Ureaplasma* spp. and *Mycoplasma* spp. PCR testing did not deliver antibiograms, these bacteria were treated with Macrolide antibiosis, either Clarithromycin or Erythromycin.

### Vaginal infection and pregnancy

The occurrence of the bacterial taxa found in women undergoing preterm birth before or after 34 weeks was compared to those women who were admitted with cervical incompetence but gave birth after 37 weeks of gestation. Mean cervical length, prolongation of pregnancy and preterm birth rates were compared in women with the two most found bacterial taxa, *Ureaplasma* spp. and *E. coli*, to women found with other bacterial colonization and unaffected women.

Antibiotic treatment was analyzed for the two most found extra-cellular species, *E. coli* and Group B streptococcus.

Data acquisition and analysis were performed with SPSS 23.0 (IBM®, 2018). Comparison between groups took place using non-parametric testing with Kruskal–Wallis test for linear variables, and post-hoc analysis was performed using Holm–Bonferroni correction. Pearson’s Chi-square test was used for nominal scaled variables.

## Results

### Clinical data

During the years 2011 and 2016, 327 patients in the second and early third trimester (until 33 weeks) were referred to the university medical center of Mainz due to cervical shortening of less than 25 mm. Mean age of these patients was 29.24 years (16–44 years), mean cervical length 17.09 mm (0–24) and mean gestational age at admission 29.17 weeks (18–33 weeks). Pregnancy could be prolonged for 7.76 weeks (3–20) after admission. The overall preterm birth rate was 28.44%; the preterm birth rate below 34 weeks was 11.01%. Table 1 summarizes baseline patient characteristics.

**Table 1:** Patient’s characteristics

Total number of patients	327
Age at admission (years)	29.24 (16–44)
Parity	
Nulliparous	203 (62.1%)
Multiparous	124 (37.9%)
Cervical length at admission	17.09 (0–24)
Gestational age ad admission (weeks)	29.17 (18–33)
Gestational age at delivery (weeks)	36.99 (25–42)
Prolongation of the pregnancy (weeks)	7.76 (3–20)
Vaginal smear	
Vaginal bacterial colonization	217 (66.36%)
No colonization	110 (33.64%)
Preterm birth rate	93 (28.44%)
< 34 weeks of gestation	36 (11.01%)
> 34 weeks of gestation	57 (17.43%)

### Microbiologic* analysis*

**Fig. 1:**
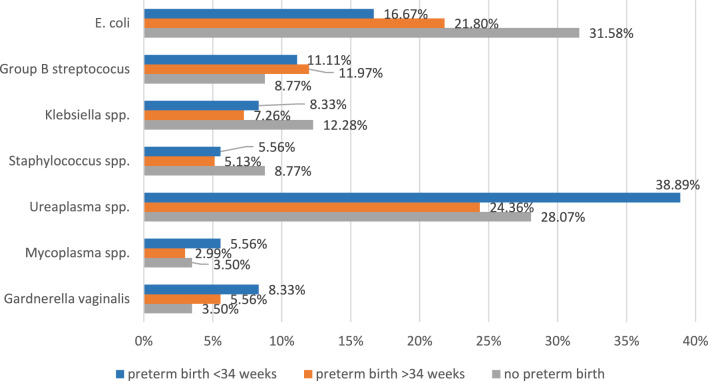
Frequency of bacterial taxa in women with cervical incompetence with and without preterm birth

Microbiologic analysis of the smears taken at hospital admission revealed seven different distinct species: *E. coli*, Group B streptococcus, *Staphylococcus* spp., *Ureaplasma* spp., *Gardnerella* *vaginalis*, *Klebsiella* spp., and *Mycoplasma* spp. The spectrum of bacterial vaginal colonization is demonstrated in Fig. 1. In total, vaginal bacterial colonization was found in 66.36% (217), and a minority of 33.64% (110) had normal vaginal flora or no bacteria at all.

### Influence of vaginal bacterial colonization on pregnancy outcome

Comparing women meeting the fate of preterm birth with those giving birth at term, no significant differences in the occurrence of vaginal colonization (treated by antibiotics) were found (Table 2). Differentiating for the various bacterial taxa, no significant differences were found likewise (Table 3).

**Table 2:** Percentage of vaginal bacterial colonization

Group	Preterm birth < 34 weeks	Preterm birth > 34 weeks	Birth on term
Bacterial colonization	11.98% (26)	17.51% (38)	70.5% (153)
No colonization/normal vaginal flora	9.09% (10)	17.27% (19)	73.63% (81)
Significance			
*X*^2^	0.656		
*P*	**0.720**

**Table 3:** Frequency of bacterial taxa in women with cervical incompetence with and without preterm birth, statistical significance

Taxa	Preterm birth < 34 weeks	Preterm birth > 34 weeks	Birth on term	Significance
*E. coli*	6/36	51/234	18/57	*X*^2^: 3.346, *p* = 0.188
Group B streptococcus	4/36	28/234	5/57	*X*^2^:0.482 *p* = 0.786
*Klebsiella* spp.	3/36	17/234	7/57	*X*^2^:1.498 *p* = 0.473
*Staphylococcus* spp.	2/36	12/234	5/57	*X*^2^:1.100 *p* = 0.577
*Ureaplasma* spp.	14/36	57/234	16/57	*X*^2^:3.320 *p* = 0.190
*Mycoplasma* spp.	2/36	7/234	2/57	*X*^2^:0.626 *p* = 0.731
*Gardnerella* *vaginalis*	3/36	13/234	2/57	*X*^2^:0.990 *p* = 0.610

### Influence and treatment of *E. coli*, *Ureaplasma *spp. and Group B streptococcus

Analyzing the three most found taxa, *E. coli*, *Ureaplasma* spp. and Group B streptococcus, we assessed the cervical length at admission, duration of pregnancy prolongation and preterm birth rates. All those parameters differed not significantly from those women with vaginal colonization comprising other bacterial taxa and those unaffected.

**Fig. 2:**
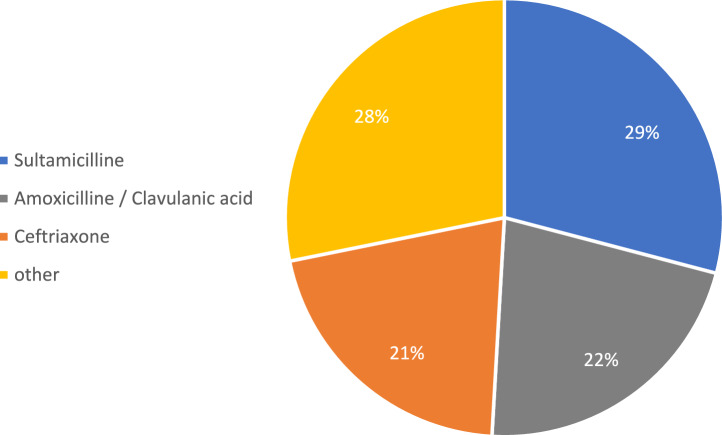
Most common treatments for *E. coli* colonization

**Fig. 3:**
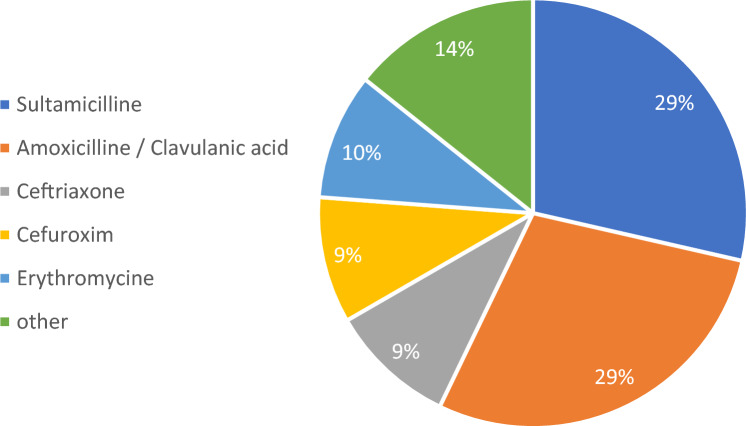
Most common treatments for GBS colonization

Interestingly enough, antimicrobial treatment with penicillins plus betalactamase inhibitors was quite common, encountering the majority of women with *E. coli* and Group B streptococcus either (Figs. 2, 3).

## Discussion

This study gives an overview over the vaginal bacterial colonization in pregnant women with cervical incompetence. In nearly two-thirds of our patients, a vaginal bacterial colonization was found. Most found species were *E. coli*, *Ureaplasma* spp. and Group B streptococcus. All patients had an antibiotic treatment according to an antibiogram. 37.5% of our patients did not show any vaginal colonization with potentially pathogenic bacteria. Irrespective of the microbiological result of the vaginal smear, the number of preterm deliveries did not differ significantly. However, a total of 93 patients (28.44%) were concerned and gave birth preterm.

It remains hard to distinguish, if the shortening of the cervix occurs because of a vaginal infection or not. Our results make seem two different conclusions possible: first: a vaginal colonization is the reason for a shortening of the cervix and an adapted antibiotic treatment is effective. Second: the vaginal bacterial colonization is not the cause of cervical insufficiency. As there are patients without any pathogenic vaginal colonization, there must be different causes for the shortening of the cervix. The etiology is supposed to be multifactorial.

In women with preterm birth before 34 weeks, *Ureaplasma* spp. was the by far most found species, and *E. coli* was the most found species in women undergoing preterm birth after 34 weeks but not for those with early preterm birth.

It is known from literature that *Ureaplasma* colonizations and infections are linked to cervical insufficiency [4], preterm labor [5], and PPROM [6], resulting in a higher incidence of preterm birth [7]. Vaginal *Ureaplasma* spp. lipoproteins are suspected to upregulate Toll-like receptor 2 (TLR 2) expression leading to induction of pro-inflammatory cytokines like IL-8 [8]. This pathway is likely to be one of the mechanisms explaining increased preterm birth rates after *Ureaplasma* spp. colonization.

In the light of this knowledge, it seems to be more surprising that there was no significant link between the diagnosis of short cervix and vaginal *Ureaplasma* spp. and preterm birth in our study population.

If it comes to vaginal infections with, for example, *E. coli*, the insight concerning potential pathogenetic pathways turns out to be less clear. As some observational studies found evidence for the association of vaginal *E. coli* colonization [9, 10] and spontaneous preterm birth especially before 34 weeks, it would be expectable that this association would also be found in our survey. But this was not the case and looking a bit more into the depth beyond the scientific level of a pure statistical correlation clinical observation unveils that there is not too much known concerning the pathophysiological pathway leading from vaginal *E. coli* colonization to preterm birth. Recently, Spencer et al. [11] succeeded to identify an inflammatory pathway after vaginal *E. coli* infection in a promising mouse model, comparable models for humans still turn out to be lacking.

Most notably, overall, none of the found bacterial taxa turned out to be “game changing” towards occurring typically in those women giving birth preterm. Overall, two explanations are plausible for these findings. First, a clear link seen between vaginal infection and preterm birth, like in the case of *Ureaplasma* spp. infections, is not known for many bacterial taxa. Second, the question arises, whether bacterial colonization in the vagina coincides with amniotic infection and inflammation as a trigger for preterm birth. As we know, inter alia, from Kayem’s and Combs’ studies [12, 13], intra-amniotic colonization may be a trigger for preterm birth, it seems doubtable, whether the finding of bacterial colonization in a vaginal fornix posterior smear correlates with intra-amniotic infection. If not, the proof of vaginal colonization in this smears may not be suitable to predict the risk of preterm birth or even the necessity of antibiotic treatment. Regarding to Daskalakis’ and Thomakos’ findings, alternatively, measuring the intra-amniotic levels of inflammatoric markers as Interleukin-6 and -18 or TNF alpha could be more accurate in detecting women at risk of preterm birth due to intra-amniotic bacterial colonization, but, on the other hand, more invasive as amniocentesis would be needed [14,15].

Antibiotic treatment without clear evidence of its benefit should be seen with extreme precaution. First, the real benefit of antibiotic treatment is quite difficult to verificate as we did not include women with evidence of bacterial colonization without antibiotic treatment. As the role of vaginal bacterial colonization is not clear, it is hard to evaluate, if antibiotic treatment was effective in patients with cervical incompetence. Second, as we identified Penicillins with Betalactamase inhibitors as treatment of first choice in even in a majority in two of the most common bacterial taxa, the antibiotic regimes used have to be seen with a critical eye as it comes to side effects. As we learned from the ORACLE trial even more than 20 years ago, Penicillins with Betalactamase inhibitors (in the case of the mentioned trial co-amoxiclav) in prophylactic treatment after PPROM were seen as harmful regarding neonatal necrotizing enterocolitis [16]. As the actual guidelines concerning the prevention and management of preterm birth do not recommend the use of these antibiotics [17–19] and in our study the treatment intention was about reducing the risk of preterm birth, we assume these types of antibiotics not suitable for women with cervical incompetence as well. There is, once more limited, but nevertheless, encouraging evidence that preterm birth is linked to distinct changes in the vaginal microbiome in early pregnancy [20, 21]. In 2019, Fettweis et al. delivered key findings on this issue in their comprehensive investigation concerning changes of the vaginal microbiome composition and preterm birth, concluding that the microbiome composition early in pregnancy may be most useful in the prediction of adverse pregnancy outcomes [22].

In the light of this knowledge, the whole approach of identifying a shortened cervix in the second and early third trimester, taking vaginal microbial smears and trying to eliminate distinct bacterial taxa as a kind of pathology-causing target turns out to be at least doubtable. Of course, as preterm birth rates were as high as 28.44%, measuring the cervix in this phase of gestation succeeded to identify patients at risk of preterm birth. But as there were almost no significant differences in preterm birth rates and prolongation of pregnancy whether distinct bacterial taxa were found or not, this approach failed to be beneficial in the prevention of preterm birth. Therefore, as a key finding of our study, we estimate that the approach of measuring cervical length and searching for germs as being causative for a shortened cervix in late second and early third trimester is not an effective approach in the prevention of spontaneous preterm birth, because it is not sufficiently linked to intra-amniotic infection, and, when it comes to prevention in the narrow meaning of the word, because it is too late. Our findings support the hypothesis that, knowing that the ascension of certain bacteria as, for example, *Ureaplasma* spp., increases the risk of spontaneous preterm birth, the identification of women at risk should take place early in pregnancy and should rest on a sophisticated analysis of the vaginal microbiome rather than identifying single bacterial taxa alone.

Without doubt, our study has several limitations. First, it was a retrospective study with a limited number of patients. Sub-group analysis was not suitable as the number of patients would get too small in sub-groups. Likewise, the fact that the difference of rates of *E. coli* colonization pregnancies with and without preterm birth was not significant may also be biased by the limited number of cases.

Nevertheless, we are convinced that our study is helpful to induce a thorough reconsideration of daily clinical practice. The worth of the microbial smear as a diagnostic tool should be the identification of a potential target of treatment in our effort to prevent preterm birth. If the process of taking smears in patients with a shortened cervix is not effective in risk management or the influence of preterm birth rates, the identification of women at risk of spontaneous preterm birth should take place earlier in pregnancy (Table 4).

**Table 4:** Diagnostic findings, prolongation of pregnancy and preterm birth rates compared after diagnosis and treatment of *E. coli*, Group B streptococcus or *Ureaplasma* spp. *: lowest level of significance after Holm–Bonferroni correction ^+^: Significance calculated by Pearson Chi-square testing

	*E. coli*	*Ureaplasma*	GBS	Other taxa	No infection
Cervical length admission (mm)	15.67	17.91	18.14	16.49	17.67
Rank	138.66	184.28	177.38	157.75	169.68
Significance*	***p* = 0.496**				
Prolongation of pregnancy (weeks)	9.33	6.93	8.19	7.74	7.08
Rank	158.69	117.96	138.75	133.33	118
Significance*	***p* = 0.084**				
Preterm birth < 34 weeks	8%	13.63%	4.76%	14.55%	9.09%
Preterm birth > 34 weeks	24%	16.67%	14.28%	14.55%	17.27%
Significance^+^	***X***^**2**^** = 7.551, *p* = 0.478**				

More research is needed concerning how the knowledge about vaginal microbiome changes in women at risk of preterm birth can be transcripted in clinical practice in the means of clinical tools and preventive treatment efforts as preterm birth still turns out to be a major burden to perinatal health [23, 24].
